# Navigation strategies as revealed by error patterns on the Magic Carpet test in children with cerebral palsy

**DOI:** 10.3389/fpsyg.2015.00880

**Published:** 2015-07-08

**Authors:** Vittorio Belmonti, Alain Berthoz, Giovanni Cioni, Simona Fiori, Andrea Guzzetta

**Affiliations:** ^1^Department of Developmental Neuroscience, IRCCS Fondazione Stella MarisCalambrone, Italy; ^2^Collège de FranceParis, France; ^3^Department of Clinical and Experimental Medicine, University of PisaPisa, Italy

**Keywords:** spatial memory, sequential memory, Corsi, MRI, brain lesion, hemiplegia, right hemisphere, reference frame

## Abstract

**Introduction:** Short-term memory develops differently in navigation vs. manual space. The Magic Carpet (MC) is a novel navigation test derived from the Walking Corsi Test and the manual Corsi Block-tapping Task (CBT). The MC requires mental rotations and executive function. In Cerebral Palsy (CP), CBT, and MC scores relate differently to clinical and lesional factors. Hypotheses of this study are: that frontal lesion specifically affect navigation in CP; that brain lesions affect MC cognitive strategies.

**Materials and Methods:** Twenty-two children with spastic CP, aged 5 to 14 years, 14 with a unilateral and 8 with a bilateral form, underwent the CBT and the MC. Errors were classified into seven patterns by a recently described algorithm. Brain lesions were quantified according to a novel semi-quantitative MRI scale. Control data were partially drawn from a previous study on 91 typically developing children.

**Results**: Children with CP performed worse than controls on both tests. Right hemispheric impairment correlated with spatial memory. MC span was reduced less than CBT span and was more selectively related to right middle white-matter and frontal lesions. Error patterns were differently distributed in CP and in typical development, and depended on right brain impairment: children with more extensive right lesions made more positional than sequential errors.

**Discussion:** In CP, navigation is affected especially by extensive lesions involving the right frontal lobe. In addition, these are associated with abnormal cognitive strategies. Whereas in typical development positional errors, preserving serial order, increase with age and performance, in CP they are associated with poorer performance and more extensive right-brain lesions. The explanation may lie in lesion side: right brain is crucial for mental rotations, necessary for spatial updating. Left-lateralized spatial memory strategies, relying on serial order, are not efficient if not accompanied by right-brain spatial functions.

## Introduction

Navigation is a complex ecological activity involving spatial cognition through body motion. A fundamental cognitive challenge facing the brain during navigation is the changing spatial relationship between one’s own body and external space. This is referred to as the *reference frame problem* (Galati et al., 2010). Sensory stimuli are first encoded in spatial reference frames centered on the sensory organs, to be then integrated by posterior parietal cortex into more global, but still *egocentric* reference frames centered on different body parts (Buneo and Andersen, 2006), and finally in an *allocentric* reference frame, i.e., one that is consistent with some environmental features and is independent of body motion (Berthoz, 1997; Burgess, 2008; Galati et al., 2010). Allocentric spatial encoding introduces a substantial computational simplification, but requires the maturation of specific neuronal populations.

The processing in egocentric and allocentric reference frames is sub-served in humans by different neural networks (reviewed in: Galati et al., 2010; see also: Galati et al., 2000; Neggers et al., 2006; Zaehle et al., 2007; Burgess, 2008) They are associated with different activation patterns within the two main visual streams (the egocentric involving dorsal, occipital–parietal stream, the allocentric requiring both dorsal and ventral areas, see Committeri et al., 2004), and are dissociable by brain lesions, as happens in hemispatial neglect (Pizzamiglio et al., 1998; Committeri et al., 2007; Grimsen et al., 2008). A striking association has been shown between side-dominance in hippocampal activation and different navigation strategies: whereas allocentric (map-based) navigation relies more on the right hippocampus, the left hippocampus is responsible for sequential egocentric navigation (Lambrey et al., 2008; Iglói et al., 2010). Noteworthy, the left vs. right-brain dichotomy is not restricted to spatial encoding: the left hemisphere, in fact, is more devoted to processing and storing serial temporal order irrespective of the type of information being stored, whereas the right hemisphere is typically more involved in global, holistic information processing, also because of micro-structural cortical and sub-cortical differences (Chance, 2014).

A long-standing developmental literature sees the different spatial strategies as relying on distinct cognitive modules. Egocentric spatial encoding is considered as appearing earlier both in phylogenesis (Wang and Spelke, 2002) and in human development (Piaget and Inhelder, 1948; Acredolo, 1978). Coherently, mature navigation skills would require allocentric encoding, instead. In fact, several authors have found flaws in children’s navigation: poor ability to take into account extrapersonal spatial relations (Overman et al., 1996) and external boundaries (Bullens et al., 2010b) until 7 years of age, strong dependence on proximal visual cues up to the age of 10 (Lehnung et al., 1998; Leplow et al., 2003), rigid reliance on previously followed paths until the age of 10 (Bullens et al., 2010a). Even the simple generation of goal-oriented locomotor trajectories has been recently found not to be fully developed until 11 years of age (Belmonti et al., 2013). Taken together, all this findings seem to confirm the Piagetian notion of a qualitative shift, occurring in the late pre-operational period, from a primary egocentric to a later allocentric stage (Piaget and Inhelder, 1948).

On the other hand, children as young as 3 years have been shown to already make use of allocentric encoding in a task of spatial judgment across perspectives (Nardini et al., 2006). Even younger children can retrieve hidden objects by using purely allocentric information (Newcombe et al., 1998; Ribordy et al., 2013). The strong qualitative and developmental opposition between egocentric and allocentric encoding seems therefore questionable. A more complex picture has been proposed by Burgess (Burgess, 2006): both egocentric and allocentric strategies would be available from an early stage, but the two would act in a complimentary fashion. Therefore, a lack of strategy-switching than a pure lack of any cognitive module can be postulated as the basis of incomplete spatial abilities in children under 10–11 years (Bullens et al., 2010a; Poirel et al., 2011). This is consistent with recent studies reporting executive functions as the main determinant of navigation skills in childhood (Purser et al., 2012).

A novel test for locomotor navigation, suitable for both children and adults, has been developed for the study of spatial orientation, visual–spatial memory, and cognitive strategies during navigation: the Magic Carpet (MC) (Meilinger et al., 2011; Demichelis et al., 2013; Belmonti et al., 2014; Perrochon et al., 2014). The MC was derived from the previous Walking–Corsi test (Piccardi et al., 2008), and both are transpositions of the traditional Corsi Block-tapping Task (CBT) to locomotor space. The CBT is probably the most widely used neuropsychological test for visual spatial memory: the subject is required to retrieve spatial sequences of increasing length by tapping on little wooden or plastic cubes (see the Section “Materials and Methods”). On the MC, instead, spatial sequences are presented in locomotor space by an electronic device and the subject is required to retrieve the sequences by walking on tiles, instead of tapping on cubes. Apart from size, the spatial layout of MC tiles is identical to that of CBT cubes.

In a recent study, we have tested the hypothesis that school-age children, in opposition to adults, would not spontaneously adopt allocentric navigational -strategies when performing on the MC, due to a lack of cognitive shifting from reaching (CBT) to navigational (MC) space. Three major findings supported our hypothesis: (a) performance (short-term memory span) increased with age comparatively more on the MC than on the CBT; (b) the amount of body rotations affected performance on the MC, not on the CBT, and comparatively more that of children than that of adults; and c) most importantly, children, unlike adults, did not differentiate error types between the two tests (Belmonti et al., 2014). Error types were classified according to a novel computerized algorithm (see the Section “Materials and Methods”).

Cerebral palsy is the most important group of disorders affecting movement and posture in childhood. Once seen as a mere motor disorder, it is now recognized that “the motor disorders of cerebral palsy are often accompanied by disturbances of sensation, perception, cognition, communication, and behavior” (Rosenbaum et al., 2005). Among the accompanying disorders, visual perceptual and visual spatial impairment is particularly relevant (Ego et al., 2015), also affecting CBT performance (Pueyo et al., 2009; Gagliardi et al., 2011). Strikingly enough, however, psychometric testing is restricted to reaching space and navigational studies involving children with CP are particularly lacking.

Locomotor navigation in CP has been recently studied for the first time using the MC methodology (Belmonti et al., 2015). That study involved a sample of 17 children and adolescents with either unilateral or bilateral spastic CP. Span scores on the CBT and the MC were studied in relationship to clinical and neuro-radiological factors. CBT span resulted significantly lower in CP, especially in bilateral forms, than in controls, while MC span did not significantly differ between groups. Preterm birth and visual disorders affected performance on the CBT but not on the MC, whereas both were related to right-hemispheric impairment (Belmonti et al., 2015).

The present study is aimed at extending our previous investigation. A larger sample was recruited in order to check the hypothesis that navigational performance is actually reduced in CP and that navigational impairment depends on lesion extension to the frontal lobes. In addition, we wanted to investigate error patterns in CP in relationship with brain lesion characteristics. Error analysis, which was not performed by Belmonti et al. (2015), allows to infer the cognitive strategies used by each subject to solve the task, as previously shown for typically developing individuals (Belmonti et al., 2014). Different navigational strategies were expected to be adopted by children with CP from those found in typical development. In fact, several strategies can be used by healthy subjects, whereas brain lesions limit the choice to a restricted range of solutions on many cognitive tasks, and this limitation can be expected also for the MC. In particular, the lesion of either the left or right hemisphere could affect specifically sequential or global spatial encoding, respectively.

## Materials and Methods

### Subjects

All patients admitted to IRCCS Fondazione Stella Maris, Pisa, from January 2013 to April 2014, were screened for the following inclusion criteria: age between 5 and 14 years, diagnosis of spastic CP, independent walking without aids, available brain MRI scans performed after 18 months of age. The final sample was of 22 subjects, as described in **Table 1**. This sample is an extension of that enrolled by Belmonti et al. (2015), which comprised 17 of the subjects (subjects 9, 13, 14, 17, and 20 did not participate in that study).

**Table 1:** Main characteristics of the sample.

Subject data					Lesion characteristics and MRI scores											Functional impairment		Corsi and Magic Carpet scores					
Subject	Age	Gender	GA	CP type	Lesion side	Lesion type	GS_HR	GS_HL	Frontal	Temporal	Parietal	Occipital	PWM	MWM	CS	GMFCS	VF	Verbal IQ	CBT	MC	Place-sparing	Path-sparing	Min/rnd.
1	6	F	30	H	R	PWM	4.5	0	1	1.5	1.5	0.5	3	1.5	0	I	0	N	3	3.33	3	1	0
2	6.17	M	38	H	L	CDGM	0	9	3	3	3	0	3	3	3	I	0	N	3.66	3	2	2	0
3	7.17	F	37	H	L	CDGM	0	5.5	1.5	2	2	0	1.5	2	2	I	0	N	5.33	3.66	3	0	0
4	7.5	M	35	H	L	CDGM	0	9	2.5	3	3	0.5	2	3.5	3	II	1	N	4	3.33	1	3	0
5	8.33	F	40	H	L	BM	0	6	2	2	2	0	0	3	3	I	0	N	4.33	3.33	3	1	0
6	8.75	F	37	H	L	CDGM	0	8	2	3	2	1	1	3.5	3.5	I	1	N	4.66	4.66	2	2	0
7	8.92	M	39	H	L	BM	0	6	2	2	2	0	0	3	3	I	0	N	4.33	4	5	0	0
8	9.33	M	40	H	R	PWM	2.5	0	0.5	1	1	0	1	1.5	0	I	0	BI	6	4.66	3	0	0
9	10	F	40	H	B	CDGM	5.5	0.5	0	1.5	3	1.5	3	2	1	I	0	N	4.66	4	4	1	0
10	11.42	F	40	H	R	PWM	4	0	1	1	2	0	1.5	2.5	0	II	2	BI	4.33	4	2	3	0
11	12.08	M	41	H	L	PWM	0	7	1.5	2	2	1.5	4	3	0	I	0	N	7.66	8	4	0	1
12	12.17	M	41	H	R	BM	5.5	0	1.5	2	2	0	0	2.5	3	I	0	BI	5.66	4	1	4	0
13	13.08	F	40	H	L	PWM	0	2.5	1	0	1.5	0	1.5	1	0	I	0	N	5.66	6	4	1	0
14	14.25	M	40	H	R	PWM	4	0	1	1	1.5	0.5	2.5	1.5	0	I	0	N	6	5.66	3	0	0
15	5.17	M	30	D	B	PWM	5.5	5.5	2	2	4	3	7	4	0	II	2	BI	2.33	2.66	0	3	0
16	7	M	34	D	B	PWM	5.5	6.5	2.5	2.5	4	3	7.5	4.5	0	I	0	N	3.66	4.33	1	2	1
17	7	F	32	D	B	PWM	6	6	3	3	4	2	7	5	0	I	1	N	4.33	4	3	2	0
18	7.67	M	30	D	B	PWM	6	6	2	4	3	3	7	5	0	I	1	N	3.66	3.66	1	2	0
19	9.33	F	33	D	B	PWM	7	7.5	3	3.5	4	4	8	6.5	0	II	1	BI	3.33	2	4	1	0
20	10.75	F	30	D	B	PWM	6	7	3	3	4	3	8	4	0	II	2	N	4.33	4.33	2	2	0
21	11.5	F	28	D	B	PWM	3.5	3.5	2	1	3	1	5	2	0	II	1	N	3	4.33	2	2	0
22	12	F	36	D	B	PWM	6.5	7	3	3.5	3.5	3.5	8	5.5	0	II	2	N	4	3.66	1	2	0

*Legend: GA, Gestational Age at birth; H, hemiplegia; D, diplegia; R, Right, L, Left, B, Bilateral; GMFCS, Gross Motor Function Classification System; VF, Visual Function; 0, no impairment, 1, mild impairment; 2, severe impairment; IQ, Intelligence Quotient; N, normal; BI, borderline intelligence; CBT, Corsi Block-tapping Task span; MC, Magic Carpet span; Min./Rnd., Minimal/Random; GS, Global Score; HR, Hemispheric Right; HL, Hemispheric Left; PWM, Periventricular White Matter; MWM, Middle White Matter; CS, Cortical–Sub-Cortical lesion*.

The study was approved by the local Ethics Committee of the IRCCS Fondazione Stella Maris, Pisa, Italy (protocol number 07/2012).

Control data were obtained in part from a sample of 91 typically developing children aged 5 to 12 years recruited for our previous study on typical development (Belmonti et al., 2014). Four healthier, typically developing children were enrolled, in order to match in age and gender four subjects whose age exceeded 12 years. From the whole control sample, a subset was extracted by age- and gender-matching controls with CP children. The matching procedure was as follows: for each CP subject, the control subject of the same gender and nearest age was selected. If two CP subjects corresponded to one control, the latter was matched with the subject nearest in age and a second control was selected to match the other subject.

### Set-Up and Procedure

**FIGURE 1:**
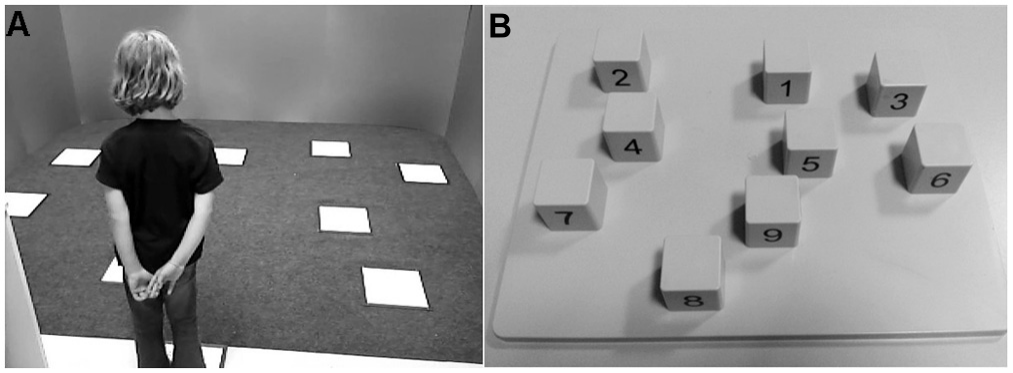
**Experimental setup. (A)** Magic Carpet. **(B)** Corsi Block-tapping task (experimenter’s view).

The CBT was performed on a plastic board (28 cm × 22.5 cm) with nine blocks on top (2.9 cm × 2.9 cm × 3 cm). This was part of BVS-Corsi standardized package (**Figure 1**).

The MC is an experimental test. It consists of 10 square tiles (30 cm × 0 cm), each containing four pressure sensors, and a central blue LED (**Figure 1**). Nine tiles are embedded within a gray carpet (310 cm × 260 cm), with the same geometric layout of CBT blocks. The tenth tile, placed outside the carpet, is the starting point. A laptop manages I/O signals. Dark-gray panels, two meter high, were placed all around the carpet, except on the starting side, so that landmarks were covered.

On both tests, the standard procedure for short-term memory assessment was applied. On the CBT, the experimenter taps a sequence of blocks, which the subject must retrieve by finger-tapping those blocks in the same order after a start signal. The procedure for the MC is identical, except that stimuli are automatically delivered by LED switching (tiles are lit up) while the subject stands on starting point, and then retrieved by the subject walking on those tiles in the same order. Sequence length identifies the level, increasing from 2 to 9. Five pseudo-random sequences per level are shown, and a level is passed when three of them are correctly retrieved, otherwise the test is terminated. Span is given by the highest level passed, plus an additional 0.33 accredited for every correct response at the highest, non-passed level. This scoring system was used in previous studies (Belmonti et al., 2014, 2015) and is aimed at increasing resolution.

### Normalization of Span Scores

Corsi Block-tapping Task and MC raw span scores were normalized by age, using the linear regression models fitted on control data. For each test, normalized span was given by:

(1) $$span_{norm}=\frac{span-\left(b_{0}+b_{1}age\right)}{SE}\;$$

where *b*_0_ and *b*_1_ are the intercept and the slope of controls’ span-by-age linear regression line, and SE is the SE of the residuals.

### Classification of Errors

Since locations were labeled by integers from 0 to 9 (0 for the starting location) on both tests, stimulus and response sequences could be coded as numeric strings and automatically analyzed by a computer algorithm, in analogy to DNA transcription errors. The methodology has been already described (Belmonti et al., 2014).

Four basic point errors were identified:

(1) *Omission*: a location present in the stimulus is absent in the response, and the number of locations in response is lower;
(2) *Substitution*: a location present in the stimulus is replaced by a different one in the response;
(3) *Insertion*: a location present in the response is absent in the stimulus, and the number of locations in response is higher;
(4) *Permutation*: two locations present in the stimulus are present in the response in inverted order.

**FIGURE 2:**
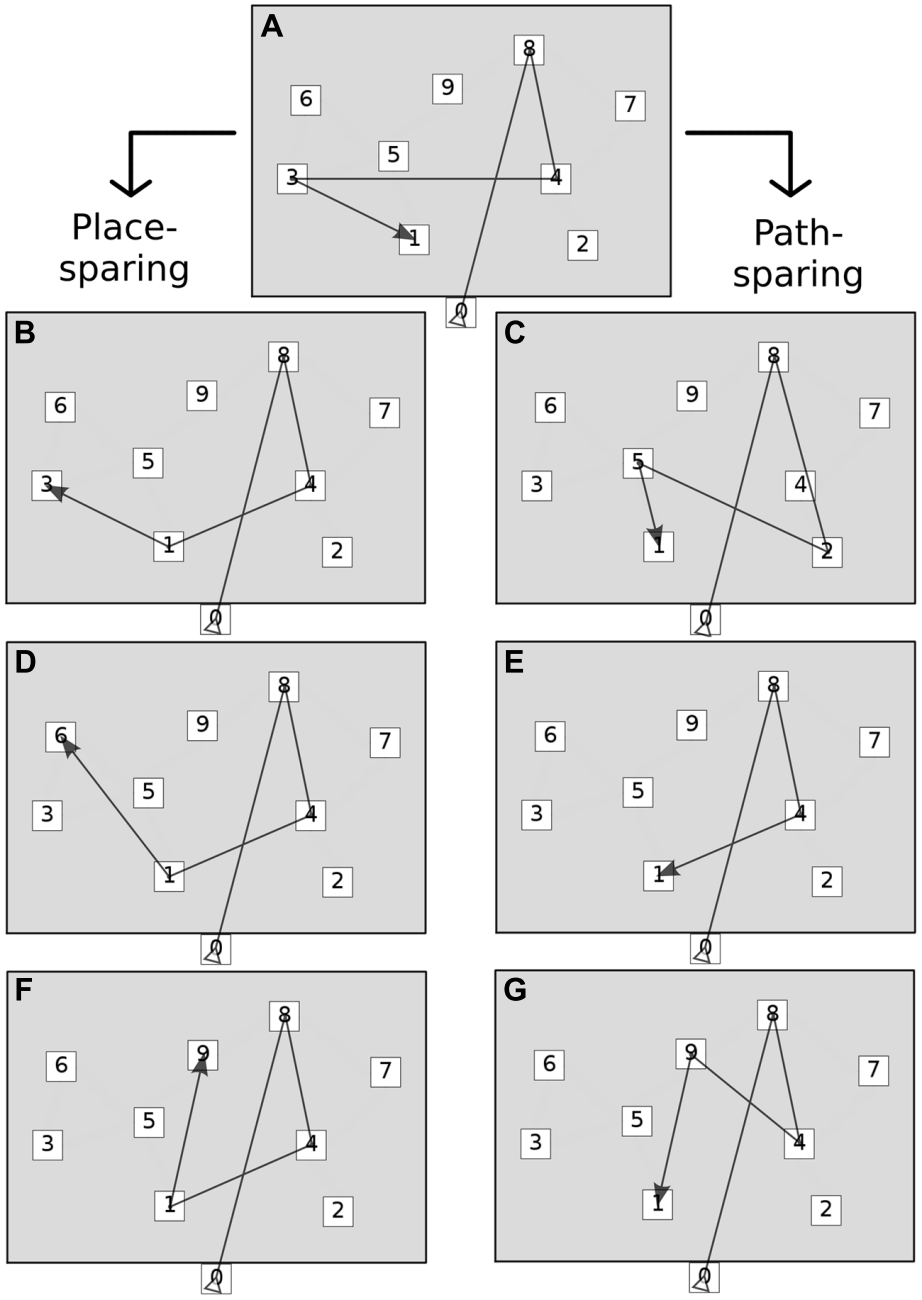
**Global error patterns.** The figure shows the two main groups of error patterns: place-sparing errors (left) and path-sparing errors (right). Minimal and random responses are not shown. See **Table 2** for the operational definition of each pattern. **(A)** Correct sequence, **(B)** Place identity (permutations only), **(C)** Path approximation (substitutions near to correct path), **(D)** Place approximation (permutations and substitutions near to correct locations), **(E)** Path shortening (omissions and possibly substitutions), **(F)** Place intrusion (permutations and substitutions far from correct locations), **(G)** Path deviation (substitutions far from correct path).

Point errors often combined giving rise to complex associations. Moreover, the analysis of numeric strings alone could not account for the spatial shaping of errors. Therefore, incorrect responses were further classified on the basis of point errors and location coordinates into seven global error patterns. The scheme originated from the observation that permutations were by far the most frequent isolated point error on both tests, leading to the hypothesis that serial order and spatial information could be dissociated and separately stored, usually to the expenses of the former. We thus hypothesized that the presence of permutations could distinguish error patterns as either *place-sparing*, i.e., preserving locations, not serial order (patterns 1 to 3), or *path-sparing*, i.e., preserving order and global path geometry, but losing spatial accuracy (patterns 4 to 6). Finally, pattern 7 included ‘minimal’ and ‘random’ responses (omission of more than half of the sequence or retrieval of less than two correct locations in correct order, respectively). The detailed classification of the seven error patterns is provided in **Table 2**. Error patterns are also depicted in **Figure 2**.

**Table 2:** Automatic classification of global error patterns: operational definitions.

**(A) Place-sparing patterns**^a^	
Error pattern 1 (*place identity*)	There are permutations with or without omissions.
Error pattern 2 (*place approximation*)	There are permutations and substitutions, with or without omissions or insertions; matching each substitution in response to a substituted counterpart in stimulus (the nearest substituted location^b^), the distance between the two never exceeds the max. threshold distance^c^; if there are insertions, each of them is within the max. threshold distance from a correct location.
Error pattern 3 (*place intrusion*)	There are permutations, plus substitutions and/or insertions not matching criteria for pattern 2.
**(B) Path-sparing patterns**^a^	
Error pattern 4 (*path approximation*)	No permutations; there are substitutions and/or insertions, with or without omissions; each incorrect location (substitution or insertion) lies within the max. threshold distance^c^ from the correct path^d^;
Error pattern 5 (*path shortening*)	There are only omissions.
Error pattern 6 (*path deviation*)	No permutations; there are substitutions and/or insertions not matching criteria for pattern 4.
**(C) Minimal or random response**	
Error pattern 7	Omissions involve half or more of the locations and/or there are not two correct locations in correct order.

*^a^First always check criteria for pattern 7: all other patterns require that omissions spare more than half of the locations *and* at least two correct locations in correct order are retrieved*.

*^b^Matching substitutions with substituted counterparts is accomplished by trying all possible permutations of the incorrect locations. The permutation that minimizes the mean distance between each substitution and its substituted counterpart provides the best matching. If there are insertions (i.e., response is longer than stimulus), these are distinguished from substitutions by trying all possible combinations of incorrect locations: the combination yielding the minimal mean distance from correct counterparts identifies substitutions, whereas the other incorrect locations are identified as insertions. This method also allows to detect permutations combined with substitutions*.

*^c^The maximum threshold distance was set to 90.14 cm on the MC and 8.50 cm on the Table Corsi; this is the maximum distance between any two adjacent locations (notably, it is distance between number 7 and 8). All couples of adjacent locations are shown in **Figure 2***.

*^d^The path is formed by all straight segments connecting two consecutive locations, starting from number 0*.

In addition to pattern classification, other features of incorrect responses were extracted: primacy effect (correct retrieval of the first location) and recency effect (correct retrieval of the last location).

### Clinical Characteristics and Brain Lesions

Basic visual function was scored as: 0 (no impairment), 1 (one of: mild acuity reduction, unilateral field defect, or strabismus), 2 (more severe or multiple disorders).

**FIGURE 3:**
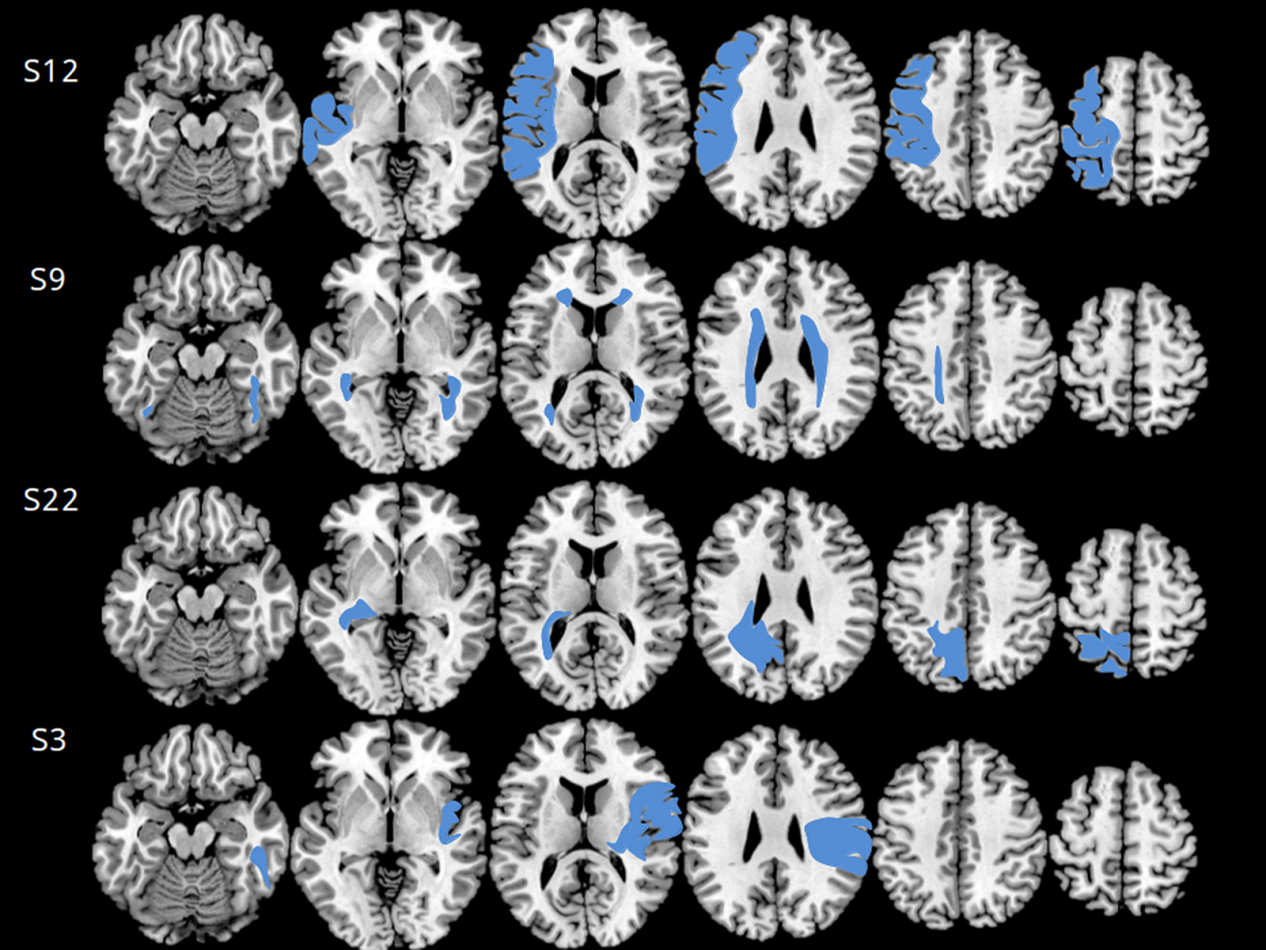
**Axial MRI scans used for scoring according to Fiori et al. (2014), relative to four subjects (subject numbers are indicated on the left).** The six axial scans best matching the scale template is selected for each subject (see reference and text for further details on the Section “Materials and Methods”). The four cases shown are different from one another with respect to lesion side, type and extension. Subject 12 had an extensive right brain mal-development involving both gray and middle white matter. The lesion extends well into prefrontal cortex. He scored 4 on the Magic Carpet, which is low for age 12, especially for a CBT score of 5.66, and made 4 path-sparing errors out of 5. Subject 9 had a typical bilateral periventricular leukomalacia, extending to middle white matter and to anterior brain regions. She scored 3.66 on the Magic Carpet and 4 on the CBT, both low scores for age 12, and made 2 path-sparing errors out of 3. Subject 22 had a cortical–sub-cortical gray- and white-matter lesion, mostly on the right side but limited to the occipital and parietal lobes. She scored 4 on the MC and 4.66 on the CBT, making only 1 path-sparing error out of 5. Subject 3 had a typical arterial stroke in the territory of the left middle cerebral artery. She scored 3.66 on the MC and 5.33 on the CBT, making only place-sparing errors. The MC score of the latter subject is in line with control data for age 7, and the gap between CBT and MC scores is probably more related to age than to the lesion.

MRI was performed using a 1.5 Tesla Magnetom (GE) scanner. T1, T2, and T2^∗^ weighted images were obtained with SE, FSE, GRE, FLAIR sequences on sagittal, axial, and coronal planes or 3D. MRI scans were taken in different epochs, but all after the age of 18 months. MRI was not necessarily performed at the same time as behavioral assessment. Structural anomalies were first classified according to Krägeloh–Mann and Horber (Krägeloh-Mann and Horber, 2007) into: Brain Mal-developments (BM), Periventricular White Matter lesions (PWM), Cortical and Deep Grey Matter lesions (CDGM), or Miscellaneous (M). Then, a novel semi-quantitative scale (Fiori et al., 2014) was applied (**Figure 3**). Anomalies are matched with a graphical template divided into six axial brain slices, plus others for basal ganglia, brainstem, corpus callosum, and cerebellum. This enables to quantify the involvement of each of three major layers (periventricular, middle, and cortical–subcortical) within each lobe: frontal, parietal, temporal, and occipital. A raw score is given to each layer on each lobe, by the number of slices where it is involved divided by the number of slices where it is represented. Raw scores are then transformed into semi-quantitative sub-scores: 0 (no involvement), 0.5 (up to half of the slices involved), or 1 (>half of the slices involved). The sum of layer sub-scores on each lobe gives the lobar sub-score, ranging from 0 to 3. The hemispheric Global Score (0–12) is the sum of lobar sub-scores on that hemisphere. The total Global Score (0–40) is the sum of right and left hemispheric scores, plus the global scores for: basal ganglia and brainstem (0–10), corpus callosum (0–3), and cerebellum (0–3). The scale has demonstrated high inter-rater reliability (intraclass correlation coefficient 0.92) and intra-rater reliability (intraclass correlation coefficient 0.91).

### Statistical Analysis

Raw span scores were compared between the two tests (CBT and MC), as well as between CP subjects and matched controls, by means of a two-way ANOVA for repeated measures with two within-subject factors (CBT vs. MC and CP vs. control). This aimed at evaluating the effects of CP on either test performance and on the within-subject difference between the two tests, i.e., to check whether CP determined better performance, relative to controls, on either the CBT or the MC. Then, in order to evaluate the effects of the various CP characteristics on performance, age-normalized CBT and MC span scores were analyzed by means of univariate ANOVA in relationship to each of the following factors: lesion side, lesion type, visual function, and GMFCS, applying Holm–Bonferroni correction to *p*-values. *Post hoc* analysis was for carried out by means of Tukey test. The effect of GA at birth was assessed by means of univariate linear regression analysis. Noteworthy, separate tests for different between-subject factors were preferred instead of a single multivariate analysis, because of the strong interaction expected among the factors studied.

The relationship between each MRI score, whose statistical distribution is unknown, and the two normalized span scores was studied by means of Spearman’s correlation.

Error analysis was conducted on the incorrect responses given by every subject at the maximum level reached (i.e., span level +1). The frequencies of isolated point errors, global error patterns, primacy, and recency effects were studied by means of appropriate X^2^ tests, in relationship to: CP vs. controls; CP side (right, left, bilateral); lesion type (PWM, CDGM, BM); and visual function impairment. In addition, the probability of making either a place-sparing or a path-sparing error was studied in relationship to each of the MRI global and sub-scores by means of univariate logistic regression (assuming a binomial distribution for the probability of making a path-sparing error).

## Results

### CBT and MC Performance in CP Children vs. Controls and in CP Sub-Groups

Raw CBT and MC span scores are reported in **Table 1**, together with the main scores of MRI classification and subject characteristics.

The first question was whether and how CP affected performance on the two tests. Two-way ANOVA for repeated measures, with test (CBT vs. MC) and CP vs. control as within-subject factors, revealed significant effects of test [*F*(1,21) = 3.98, *p* < 0.001], CP vs. control [*F*(1,21) = 13.35, *p* = 0.001], and of the interaction factor [*F*(1,21) = 5.58, *p* = 0.028] on span scores. Span was on average higher in controls than in CP and on the CBT than on the MC test, as expected from previous studies (Belmonti et al., 2014, 2015). CP children had a mean CBT span of 4.45 (SD = 1.22) and a mean MC span of 4.12 (SD = 1.24), whereas controls had a mean CBT span of 5.50 (SD = 1.14) and a mean MC span of 4.62 (SD = 0.86). The significant effect of interaction [*F*(5,58), *p* = 0.028] is a new and unexpected result, meaning that span difference between CBT and MC was on average greater for controls than for CP children, or, in other words, that CP children performed much worse than controls on the CBT while only a little worse on the MC.

Then, performance on either test was studied in relation to the main CP characteristics, taking one between-subject factor at a time. As far as lesion side is concerned, univariate ANOVA showed a significant difference on the age-normalized CBT among the four groups: bilateral, left-sided, right-sided lesions, and controls [*F*(3,40) = 9,175, adj. *p* < 0.001]. Tukey *post hoc* test revealed significant differences between children with bilateral lesions and controls (*p* < 0.001), and between bilateral and left lesions (*p* = 0.016). Mean age-normalized CBT span was –2.13 (SD = 1.10) in children with a bilateral lesion, –1.15 (SD = 1.00) in children with a right lesion, and –0.49 (SD = 1.06) in those with a left lesion. On the MC test, no significant difference was found among the four groups [*F*(3,40) = 2.861, adj. *p* = 0.098]. Children with a bilateral lesion had a mean age-normalized MC span of –1.35 (SD = 1.54), children with a right lesion –0.99 (SD = 0.91), and those with a left lesion 0.04 (SD = 2.03).

The effect of lesion type, as defined in the Methods section (PWM, CDGM, or BM), was then assessed. Univariate ANOVA showed a significant difference on the age-normalized CBT span among the four groups [*F*(3,40) = 5.73; adj. *p* = 0.012]. Tukey test revealed a significant difference only between children with a PWM lesion and controls (*p* = 0.001). Mean age-normalized CBT span in children with a PWM lesion was –1.53 (SD = 1.43), in children with a CDGM lesion was –0.65 (SD = 0.81), and in children with a BM lesion was –1.06 (SD = 0.16). No significant difference for lesion type was found on the MC test [*F*(3,40) = 1.66, adj. *p* = 0.19]. Mean age-normalized MC span in children with a PWM lesion was –0.56 (SD = 2.01), in children with a CDGM lesion was –0.78 (SD = 0.73), and in children with a BM lesion was –1.51 (SD = 0.70).

Visual function impairment had a significant effect on age-normalized CBT span [*F*(2,41) = 10.94, adj. *p* < 0.001]. Tukey test showed significant differences between children with grade-1 visual function impairment and controls (*p* = 0.007), as well as between children with grade-2 visual function impairment and controls (*p* = 0.001). On the contrary, visual function had only a nearly significant effect on age-normalized MC span [*F*(2,41 = 4.36, adj. *p* = 0.06].

We also performed Spearman correlation within the CP group, and found that visual function impairment significantly and inversely correlated with age-normalized CBT span (ρ = –0.58, *p* = 0.005) as well as with MC span (ρ = –0.46, *p* = 0.03).

Gross-motor function impairment had a significant effect on age-normalized span scores obtained on both the CBT [*F*(2,41) = 17.16, adj. *p* < 0.001] and the MC [*F*(2,41) = 7.05, adj. *p* = 0.012]. Tukey test revealed significant differences between children with a GMFCS level II and controls (CBT: *p* < 0.001; MC: *p* = 0.002) and between GMFCS level II and level I (CBT: *p* = 0.001; MC: *p* = 0.01).

GA at birth proved to be a determinant of age-normalized CBT span (ß = 0.15, *p* = 0.01, *R*^2^ = 0.27), whereas it did not influence MC span (*p* = 0.28, *R*^2^ = 0.06).

As in typical development, age proved to be linearly related to both CBT span (ß = 0.28, *p* = 0.004, *R*^2^ = 0.35) and MC span (ß = 0.30, *p* = 0.003, *R*^2^ = 0.37). Gender, instead, had no significant effect, neither on the CBT [*t*(19.9) = 1.60, *p* = 0.13] nor on the MC [*t*(16.3) = 1.25, *p* = 0.23].

### Relationship between MRI Scores and Span Scores

The relationship between each MRI score and the two age-normalized span scores was studied by means of Spearman’s correlation. All MRI correlations are reported in **Table 3**.

**Table 3:** Relationships between span scores, Magic Carpet error patterns, and some clinical and MRI features.

		GA	VF	GS	GS_HR	GS_HL	HSS_RPV	HSS_RM	HSS_RCS	RO	RP	RT	RF
CBT age-normalized	ρ	**0.52**	**–0.58**	–0.19	**–0.55**	0.14	**–0.65**	**–0.50**	0.11	**–0.62**	**–0.53**	**–0.40**	**–0.53**
	p	**0.013**	**0.005**	0.40	**0.007**	0.54	**<0.001**	**0.017**	0.64	**0.002**	**0.012**	**0.062**	**0.012**
MC age-normalized	ρ	0.24	**–0.46**	–0.07	–0.39	–0.05	–0.25	**–0.45**	–0.26	–0.24	–0.40	–0.34	–0.40
	p	0.28	**0.031**	0.77	0.08	0.83	0.26	**0.033**	0.25	0.28	0.062	0.12	0.066
Path-sparing error prob.	ρ	–0.10	**0.92**	0.08	**0.16**	0.04	0.16	**0.40**	0.49	0.40	0.40	0.55	**0.81**
	p	0.06	**0.003**	0.13	**0.05**	0.61	0.24	**0.05**	0.13	0.22	0.08	0.08	**0.03**

*Significant correlations are in bold*.

*CBT, Corsi Block-tapping Task span; MC, Magic Carpet span; GS, Global Score; HR, Hemispheric Right; HL, Hemispheric Left; HSS, Hemispheric Sub-Score; RPV, Right Periventricular; RM, Right Middle; RCS, Right Cortico-subcortical; RO, Right Occipital; RF, Right Frontal; RT, Right Temporal; GA, Gestational Age at birth; VF, Visual Function*.

Global scores (GS, GS-HR, and GS-HL) indicate the amount of impairment of the whole brain, the right and the left hemisphere, respectively. Total Global Score did not correlate with either CBT or MC normalized span scores. Right hemispheric Global Score (GS-HR) significantly and inversely correlated with age-normalized CBT span (ρ = –0.55, *p* = 0.007) and nearly significantly with age-normalized MC span (ρ = –0.39, *p* = 0.075). Left hemispheric Global Score (GS-HL) did not correlate with either CBT or MC span scores.

Hemispheric sub-scores indicate the amount of impairment of right and left periventricular white matter (HSS-RPV and HSS-LPV, respectively), of right and left middle white matter (HSS-RM and HSS-LM, respectively), and of right and left cortex-sub-cortical white matter (HSS-RCSC and HSS-LCSC, respectively). A strong correlation was found between the hemispheric sub-score for right periventricular white matter (HSS-RPV) and normalized CBT span (ρ = –0.65, *p* < 0.001), whereas HSS-RPV did not correlate with normalized MC span (ρ = –0.25, *p* = 0.26). HSS-RPV was also negatively related to GA at birth (ρ = –0.64, *p* = 0.001). The hemispheric sub-score for right middle white matter correlated with both normalized CBT (ρ = –0.50, *p* = 0.02) and MC span (ρ = –0.45, *p* = 0.03). The hemispheric sub-score for right cortical–subcortical white matter correlated with normalized CBT span (ρ = –0.45, *p* = 0.04), not with MC span (ρ = –0.014, *p* = 0.95).

Lobar scores indicate the amount of impairment of right and left occipital (RO and LO), parietal (RP and LP), temporal (RT and LT), and frontal (RF and LF) lobes. Age-normalized CBT span significantly correlated with RO (ρ = –0.62, *p* = 0.002), RP (ρ = –0.53, *p* = 0.01), and RF (ρ = –0.53, *p* = 0.01), nearly significantly with RT (ρ = –0.40, *p* = 0.06). Age-normalized MC span correlated nearly significantly with RP (ρ = –0.40, *p* = 0.06), and RF (ρ = –0.40, *p* = 0.07), not with RO nor with RT. Neither span score correlated with any of the left lobar scores.

None of the other MRI sub-scores significantly correlated with any functional score.

### Effects of CP Characteristics on MC Errors

The frequencies of global error patterns on the MC were studied by means of appropriate X^2^ tests, in relationship to: lesion type; lesion side (right, left, bilateral); gross-motor function impairment (GMFCS); visual function impairment. A total of 90 incorrect responses out of 110 trials were analyzed.

Children with a bilateral lesion made a total of 14 place-sparing, 16 path-sparing, and 1 random error. Children with a right unilateral lesion made a total of 16 place-sparing, 9 path-sparing, and 0 random errors. Children with a left unilateral lesion made a total of 24 place-sparing, 9 path-sparing, and 1 random error. Since random/minimal responses were only 2, they were excluded from the following analyses.

The frequency distribution of place- and path-sparing errors nearly significantly differed between bilateral and unilateral lesions [χ^2^(1) = 3.26, *p* = 0.07], whereas it did not differ between right and left unilateral lesions [χ^2^(1) = 0.18, *p* = 0.67].

The frequency distribution of place- and path-sparing errors significantly differed according to GMFCS, with children at GMFCS level I making a place-sparing error in 70% of the trials and children at GMFCS level II making a place-sparing error in only 43% of the trials [χ^2^(1) = 4.84, *p* = 0.03].

The frequency distribution of place- and path-sparing errors significantly differed also according to visual function impairment [χ^2^(2) = 9.66, *p* = 0.008]. Children without visual function impairment made a place-sparing error in 75%, those with a mild impairment in 52%, and those with a severe impairment in only 33% of the trials.

Gestational age at birth had a significant effect on error pattern: logistic regression analysis revealed that the higher the GA at birth, the more the probability of making a place- vs. a path-sparing error (1^st^ order term = 0.10, *p* < 0.05).

Importantly, error patterns were also related to MC span, i.e., the higher the span, the higher the probability of making a place-sparing vs. a path-sparing error, as revealed by logistic regression (1st order term = –0.45, *p* = 0.04).

### Effects of MRI Scores on MC Errors

Finally, the probability of making either a place-sparing or a path-sparing error was studied in relationship to each of the MRI scores by means of logistic regression.

Univariate logistic regression performed on each of the MRI scores revealed that only right hemispheric global score (GS-RH), right hemispheric sub-score for middle white matter (HSS-RM), and right frontal lobar score (RF) had a statistically significant effect on error pattern, all determining an increased probability of making a path-sparing instead of a place-sparing error (GS-HR: 1^st^ order term = 0.16, *p* < 0.05; HSS-RM: 1^st^ order term = 0.40, *p* < 0.05; RF: 1^st^ order term = 0.80, *p* = 0.03).

**FIGURE 4:**
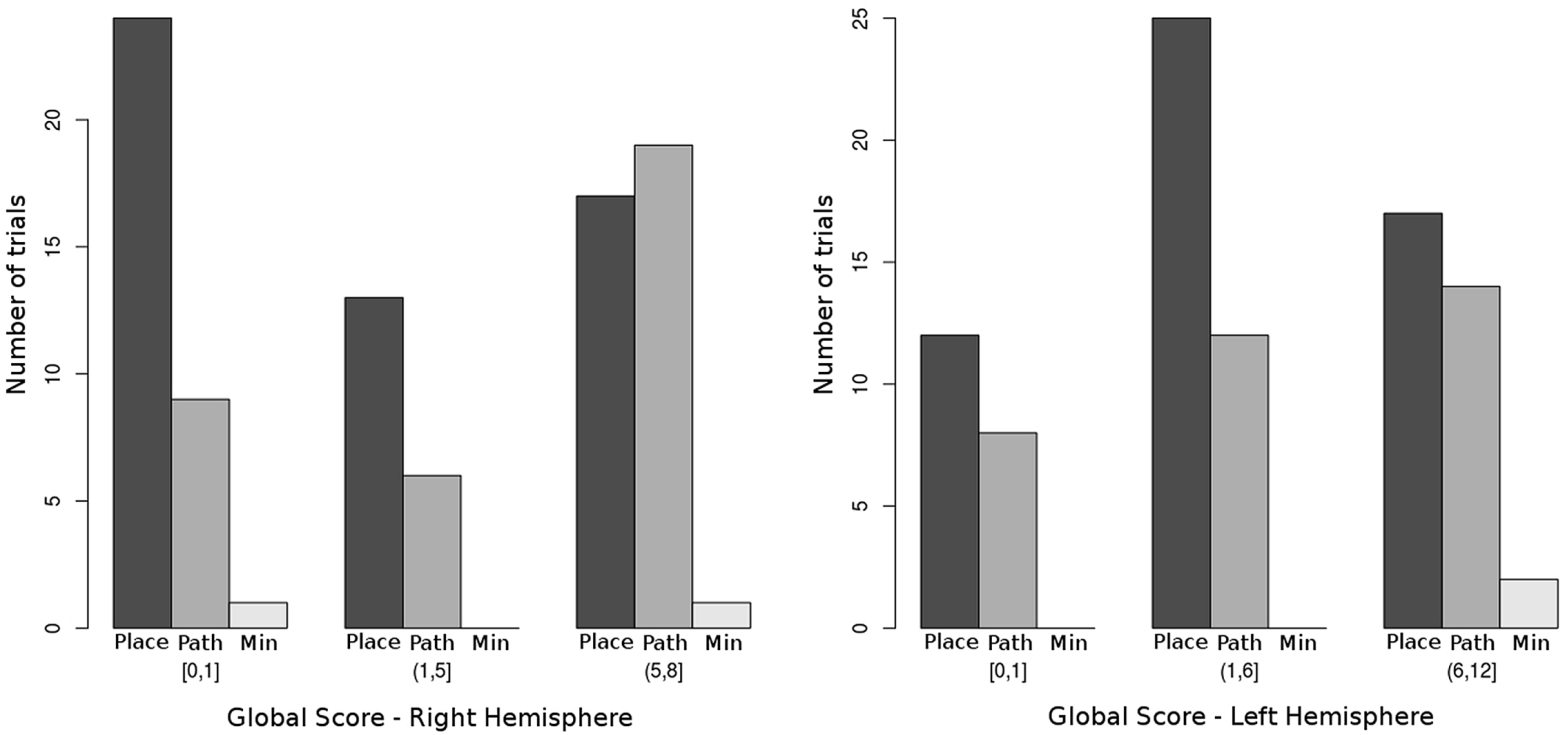
**Distribution of the three main global error categories by right hemispheric and left hemispheric impairment.** Bars indicate the raw number of trials presenting with each of the global error patterns. Place = place-sparing errors (patterns 1 to 3), Path = path-sparing errors (patterns 4 to 6), Min = minimal and random errors (pattern 7). Notice that the probability of making a path-sparing error significantly increases when the global score for right hemispheric impairment (GS-HR) increases (see **Table 3**). The relationship with left hemispheric impairment is not significant.

In **Figure 4**, the frequency distribution of the two main categories of global error patterns in relationship to GS-HR, HSS-RM and RF is shown.

## Discussion

The present study has extended our previous investigation on locomotor navigation in cerebral palsy by recruiting a larger sample, widening the analysis of span scores in relationship to several clinical and MRI characteristics, and adding the analysis of MC errors in order to study the cognitive spatial strategies employed for navigation.

### The Impairment of Spatial Memory in Reaching and Navigational Space Depends, to a Different Extent, on Lesion Extension in the Right Hemisphere

As expected, overall spatial memory performance was on average lower in CP than in controls. However, and unexpectedly, spatial memory impairment was less pronounced in navigational than in reaching space. CP children, as a group, seem to suffer from a prominent deficit of short-term visual spatial memory, which is even worse for those with a bilateral brain lesion. In comparison, navigation skills as assessed by the MC test appear relatively spared. At least two reasons can be envisaged. One probably lies in the fact that controls, i.e., typical school-age children, perform far better in reaching than in navigational space, because the latter requires advanced mental rotation skills (Belmonti et al., 2014). Therefore, navigation is a relatively underdeveloped function in the age range tested, and this may limit the negative effects of brain lesions on performance.

The second reason lies in the very matter of the present study, that is the different relationship between lesional features and spatial memory in navigational vs. reaching space. In fact, the extent of right hemispheric impairment, and in particular of the damage to right periventricular white matter, proved to be a strong determinant of CBT performance, whereas navigation seems to be affected only when the lesion extends to central white matter and/or to the right frontal lobe. Taken together, these findings point to a major effect of right brain impairment on both short-term visual spatial memory in reaching space and navigation, with a relative sparing of the latter when the lesion is limited to posterior periventricular white matter.

### Prior Evidence on Spatial Disorders and Right-Brain Lesions in Adults

The relationship between the right (or better, non-dominant) hemisphere and spatial cognition is well established in adults. Several right-lateralized networks have been described or postulated as underlying spatial perception, cognition and memory. Recently, a lateralization of dorsal and ventral visual stream functions (space and object perception, respectively) has been shown in adults with traumatic brain injury: visual–spatial functions are associated with right brain dominance and are particularly dependent on the integrity of the right inferior parietal lobule (Schintu et al., 2014). Not only perception, but also visual–spatial imagery, involving dorsal frontal–parietal networks, is lateralized to the non-dominant hemisphere in healthy adults (Sack and Schuhmann, 2012). The association between space and right brain goes beyond the visual system: vestibular functions related to self-motion perception are lateralized to right parietal–temporal areas (Dieterich et al., 2003).

Hemispatial neglect is probably the best studied spatial disorder associated with acquired right-brain damage. Right hemispheric stroke is responsible for a variety of hemispatial neglect syndromes (see for a review: Adair and Barrett, 2008), involving either personal or extrapersonal space (Committeri et al., 2007), and either egocentric or allocentric reference frames (Pizzamiglio et al., 1998; Grimsen et al., 2008; Galati et al., 2010). By contrast, neglect syndromes are seldom associated with left-brain damage and, when so, a right hemispheric dominance is usually found (Suchan et al., 2012). Importantly, for the scope of the present study, patients with hemispatial neglect also suffer from poor navigational performance (Guariglia et al., 2005). Finally, adult patients with a right hemispheric stroke score on average lower on the Walking–Corsi test than left-damaged patients (Piccardi et al., 2011). In the latter study, however, group differences related to lesion side were found only for long-term span, not for short-term span, and only on the Walking–Corsi, not on the CBT.

### Lesion Side and Spatial Disorders in Childhood: A Debated Relationship

The relationship between lesion side and spatial disorders is less clear and still debated in CP literature. Several accounts of hemispatial neglect in children with perinatal stroke do exist (Laurent-Vannier et al., 2003, 2006; Trauner, 2003; Thareja et al., 2012), but both right- and left-sided lesions are reported to cause spatial attentional disorders in children. However, whilst right-brain lesions are associated with an adult-like left-space neglect syndrome, left-brain lesions seem to determine milder and bilateral spatial inattention (Thareja et al., 2012). The incidence of spatial neglect in children with left hemispheric stroke is unknown, but seems lower than in those with right hemispheric stroke (Laurent-Vannier et al., 2006). The poorly specific visual–spatial disorders reported in children with early left-brain damage (Vargha-Khadem et al., 1994; Muter et al., 1997) have been explained with the *crowding* hypothesis: language shifting to the right hemisphere would reduce the neural resources for originally right-lateralized functions (Lidzba et al., 2006). It is unknown whether the spatial disorders described so far in right hemiplegic CP can be responsible for difficulties in navigation. In the present study, we did not find any.

### Short-Term Sequential Spatial Memory: An Early Right-Lateralized Function with Limited Plasticity?

The present study thus confirms in a larger sample our first demonstration of a specific correlation between the extent of right-brain damage and spatial disorders in children with CP (Belmonti et al., 2015). No previous study on spatial functions has ever quantified the impairment of either hemisphere in children with different CP sub-types. The absence of correlation between left-brain damage and spatial skills is consistent with prior studies on the *crowding* effect in right hemiplegia, demonstrating that CBT performance does *not* correlate with lesion size, but only with the extent to which language is shifted to the right hemisphere (Lidzba et al., 2006). Those studies indicate that short-term spatial memory is early lateralized to the right hemisphere, that left-brain impairment plays in its regard only an indirect role, and that the right hemisphere is always crucial for its development, as confirmed by our findings. This would mean that short-term spatial memory has particularly limited plasticity and suffers from any damage or overuse of the brain areas that are genetically destined to its deployment. Further investigation is needed to test this hypothesis, including functional neuroimaging studies involving subjects with lesions of different side, type, and timing.

Another possible explanation for the striking role of right-brain impairment in our study lies in the nature of the lesion. It is well known that perinatal ischemic stroke occurs much more commonly to the left than to the right hemisphere. As a result, right hemispheric lesions are mostly periventricular white-matter ones or brain mal-developments, not strokes. This situation is well represented in our sample. This means that lesion side is strongly related to lesion type and the possibility that spatial skills are sensitive to the latter rather than to the former cannot be ruled out.

### A Specific Role for Frontal Lesions in CP Navigation?

The fact that navigational performance, with respect to visual–spatial memory, is more selectively dependent on the integrity of right middle white matter (*centrum semiovale*) and of the right frontal lobe, whereas it does not correlate with posterior periventricular lesions, adds to the specificity of navigation as a spatial skill. We did not find a double dissociation between visual–spatial memory and navigation in our sample, but navigation was spared in several children with a visual–spatial deficit due to posterior deep white-matter lesions. The role of prefrontal areas and executive functions in navigation is well established in the literature (Maguire et al., 1998; Burgess et al., 2001; Pine et al., 2002; Spiers and Maguire, 2006). In typically developing children, executive functions seem even more relevant to navigation: in fact, they are the most important factor determining navigational performance at school age (Purser et al., 2012). In our prior study on typical development, we have shown that school-age children are unable to mentally switch from manual space to navigation space, as revealed by the effects of rotations and error patterns (Belmonti et al., 2014). In CP, a correlation between visual navigation (solving paper mazes) and the anterior extension of periventricular leukomalacia was found by Pavlova et al. (2007). In addition to mental shifting, working memory is another sub-component of executive function that could play a crucial role in both the CBT and the MC tasks. Noteworthy, spatial working memory is thought to be strictly related to motor learning and is particularly affected by preterm birth (Jongbloed-Pereboom et al., 2012). In summary, it comes as no surprise that navigational performance is related to more anterior lesions and that posterior lesions, which are also mostly periventricular in our sample, have a more limited impact on navigation. However, further investigation, comprising extensive neuropsychological testing, is needed to clarify the differential contribution of short-term spatial memory, mental shifting and working memory to such a complex task as the MC.

### The Role of Preterm Birth, Visual Function, and Gross-Motor Function

Preterm birth had a major effect on CBT span and a much less pronounced, not significant effect on MC span. The relationship between preterm birth and visual–spatial deficits is well established in CP neuropsychology (Atkinson et al., 2008; Ego et al., 2015). The lesions most often found in preterm-born children are PWM ones, but a more diffuse, micro-structural white matter damage is known to be responsible for reduced non-verbal intelligence in highly preterm babies, whether with or without lesions (Counsell et al., 2008). Therefore, preterm birth itself, irrespective of lesion type, may play a crucial role in determining visual–spatial dysfunction, as it seems to be confirmed by our results. Navigation, on the other hand, seems to be not as sensitive as visual–spatial memory to diffuse white-matter damage due to preterm birth, unless a lesion occurs.

The impact of visual function and gross-motor function on both visual–spatial memory and navigation is not to be underestimated. In this regard, however, clinical severity seems to play a non-specific, though important, role in determining a general reduction of spatial functions in both reaching and locomotor spaces.

### Poor Navigational Performance in CP is Associated with a Maladaptive Sequential Strategy

In our previous study on typical development (Belmonti et al., 2014), a strong preeminence of place-sparing errors over path-sparing ones was found on both the CBT and the MC test. Only in adults and on the MC, the frequency of path-sparing errors almost equaled that of place-sparing ones. In other words, path-sparing errors are a sign of more advanced navigational strategies in typical development, being associated with a higher age and higher span scores. The picture is completely reversed in children with CP. The frequency of path-sparing errors increases with the extent of right hemispheric impairment, becoming even higher than that of place-sparing ones in children with very extensive right lesions (**Figure 4**). This means that path-sparing is associated with poorer performance in CP. Therefore, the meaning of MC error patterns must be different in CP than in typical development.

Firstly, it should be considered that a small number of locations can be visually monitored during retrieval. As a consequence, very short sequences (up to 3–4 locations) do not necessarily require any strategy for coping with body rotations. Only when sequences get longer and visual monitoring becomes harder or impossible, the subject has to activate a strategy to cope with the changing viewpoint. As extensively explained elsewhere, two main spatial strategies have been described: either locations are stored in an egocentric reference frame and updated through self-motion (egocentric updating strategy), or they are encoded in allocentric coordinates from the very start (allocentric encoding strategy) (Belmonti et al., 2014). The latter strategy is more effective and less affected by body rotations, but it requires a cognitive switch to an allocentric spatial reference frame that is not fully accomplished by children under 10 (Bullens et al., 2010a). However, the point here is that subjects with a span of 3 or less do not probably need either of the two strategies, but can rather rely on visual monitoring.

The explanation above does not account for the fact that CP children with large right-brain lesions make path-sparing errors, instead of place-sparing ones like their healthy peers. Why is serial order so strongly preserved by these children? Why do they not store the single locations separately, so as healthy children seem to do? A possible explanation lies again in lesion side. The two hemispheres treat spatial information differently. The right hemisphere is implied in global, simultaneous encoding of spatial patterns, whereas the left hemisphere, though it is the non-dominant one for spatial skills, sub-serves some sequential aspects of spatial encoding. For instance, a sequential egocentric navigational strategy has been associated with activation in human left hippocampus, whereas the right hippocampus contributes much more to allocentric, i.e., map-based, navigation (Lambrey et al., 2008; Iglói et al., 2010). Therefore, if it is true that right-lateralized spatial skills do not easily shift to the contra-lateral hemisphere, children with large right-sided lesions might be forced to use a maladaptive sequential strategy relying on left spatial brain networks. The necessary developmental stage of egocentric encoding of separate locations may be prevented forever by such lesions, thus disrupting the typical developmental course of navigation strategies.

### Future Directions

Navigation is a fundamental daily activity that does not rely only on visual–spatial skills, but requires spatial encoding in multiple reference frames, strategies to cope with viewpoint changes, and advanced executive functions to manage strategy and reference-frame switching. Standard neuropsychological protocols do not assess locomotor navigation and none of the available visual–spatial tests can compare with the complexity of a real navigation task. Thanks to the Magic Carpet and other similar attempts, we are just beginning to appreciate the existence and relevance of navigation disorders in childhood. The detection of navigation disorders in Cerebral Palsy is of utmost importance for the development of habilitative and educational programs. Adaptive behavior, autonomy, and participation during adolescence and adulthood may be significantly reduced by long-standing, often undetected difficulties in spatial orientation and navigation.

Further studies are needed to ascertain the prevalence, severity and clinical implications of navigation disorders in the CP population. Moreover, the exact neuropsychological deficits and neural networks involved should be better determined and their plasticity tested by means of functional brain imaging, electrophysiological, and longitudinal case-control studies. Several questions remain unanswered, in fact: is there a critical period for the acquisition of navigation skills? To what extent and until when can navigation networks be reorganized after a brain lesion? Cannot they really shift to the contra-lateral hemisphere, and would it be desirable? How can we foster reorganization and plasticity? We have just made a little step on a brand new field of clinical research.

## Conflict of Interest Statement

The authors declare that the research was conducted in the absence of any commercial or financial relationships that could be construed as a potential conflict of interest.
